# Manganese Deficiency Causes Testicular Developmental Disorders, Blood–Testis Barrier Damage, and Spermatogenesis Disruption via Nrf2-Mediated Oxidative Stress

**DOI:** 10.3390/nu17183007

**Published:** 2025-09-20

**Authors:** Dianyi Peng, Fuqing Feng, Heng Yin, Jianfei Zhao, Shanchuan Cao, Jingbo Liu

**Affiliations:** College of Life Sciences and Agri-Forestry, Southwest University of Science and Technology, Mianyang 621010, China; pediany@163.com (D.P.); 18281650621@163.com (F.F.); yinheng@swust.edu.cn (H.Y.); zhaojf@swust.edu.cn (J.Z.); shanchuancao@swust.edu.cn (S.C.)

**Keywords:** manganese deficiency, testis, blood-testis barrier, sperm quality, oxidative stress

## Abstract

**Background**: Manganese (Mn) is a trace element essential for multiple physiological and biological processes. The testis plays a key role in male reproduction by producing sperm and synthesizing male hormones. This study investigates how Mn deficiency affects testicular development, spermatogenesis, and the blood–testis barrier (BTB), and evaluates associated variations in oxidative stress to explore potential mechanisms. **Methods**: A Mn-deficient diet was used to induce Mn deficiency in mice, with MnCl_2_ administered via intraperitoneal injection. Mn levels in testicular tissue were measured by atomic absorption spectrometry. Testis and sperm morphology were assessed by H.E. and sperm staining. BTB markers were analyzed using immunofluorescence, Western blot, and qPCR. Oxidative stress was evaluated biochemically. Nrf2 pathway changes were detected by qPCR and Western blot. **Results**: The results indicated that Mn deficiency dramatically decreased the testicular index, caused abnormal testicular tissue structure, and significantly decreased Johnsen’s score. At the same time, sperm density and motility were significantly reduced, and the sperm deformity rate was significantly increased. In addition, the BTB function was impaired, as indicated by the significantly down-regulated expression of tight junction proteins including *Occludin*, *ZO-1*, *JAM-A*, and *Claudin-11*. As the oxidative stress levels increased, the mRNA and protein expression levels of molecules (including *Nrf2* and *HO-1*) related to the Nrf2 signaling pathway were significantly down-regulated, while its inhibitor *Keap1* exhibited significantly up-regulated expression. Notably, after supplementing MnCl_2_, all the above abnormal indicators were significantly improved. **Conclusions**: Mn deficiency can lead to testicular tissue damage, decreased sperm quality, and BTB dysfunction, and the potential mechanism is probably closely associated with the increase in the oxidative stress level mediated by the Nrf2 pathway.

## 1. Introduction

Manganese (Mn), as one of the most abundant metallic elements in the Earth’s crust, is ubiquitously distributed in the natural environment and serves as an essential trace mineral for the normal growth, development, and maintenance of cellular homeostasis in humans and a wide range of other organisms [1,2,3]. Mn serves as a critical cofactor for a variety of enzymes, including oxidoreductases, hydrolases, transferases, isomerases, lyases, and ligases, and it also activates DNA and RNA polymerases, facilitates the synthesis of proteins, hormones, and vitamins, and plays a crucial role in genetic information transfer, growth, development, reproduction, bone formation, and central nervous system function [4,5,6]. Overall, Mn takes part in many physiological processes, such as regulation of glucose metabolism, reproductive function, digestive processes, bone development, nervous system function, and immune system activity.

Mn is a trace element crucial for the reproductive system and plays a key role in reproductive health and pregnancy outcomes. Early animal research has demonstrated that Mn deficiency in the diet may lead to disrupted ovulation, testicular degeneration, and elevated offspring mortality rates. Prolonged feeding of a Mn-deficient diet in cows results in suppressed estrus, characterized by the declined conception rate, rising abortion rate, and lower birth weight of calves [7]. Mn deficiency in sows is associated with disrupted estrous cycles, recurrent abortion, and elevated stillbirth rates [8]. Furthermore, severe Mn deficiency in male animals can result in infertility and reduced libido, accompanied by seminiferous tubule degeneration, absence of sperm, and accumulation of degenerative cells in the epididymis. Additionally, Mn deficiency-induced male reproductive dysfunction may be linked to the suppression of cholesterol and its precursor synthesis, which in turn limits production of sexual hormones [9]. Another study has indicated that Mn deficiency weakens the regulatory effect of adrenaline on sugar metabolism, leading to the arrest of the development of the seminiferous tubules in male poultry and a decline in the ovulation capacity of female poultry [10]. However, the mechanisms underlying the influences of Mn on reproductive health have not been comprehensively elucidated.

During aerobic metabolism, living organisms maintain continuous production of reactive oxygen species (ROS). When the generation rate of ROS (especially O_2_^−^) in the body exceeds the clearance capacity, it leads to an oxidative stress state [11]. The organism defends against oxidative stress through its antioxidant defense system by eliminating ROS, wherein antioxidant enzymes serve as key mediators that effectively scavenge or convert ROS, thereby mitigating oxidative damage. As an essential cofactor, Mn plays a critical role in this protective mechanism by contributing to the structure and function of several vital antioxidases, such as pseudocatalase, Mn superoxide dismutase (Mn-SOD), and the Oxygen-Evolving Complex [12]. Mn-SOD is predominantly localized in the mitochondrial matrix, where it functions to scavenge O_2_^−^ generated by the mitochondrial respiratory chain. This enzyme is essential for safeguarding mitochondrial integrity and function against oxidative damage, with its catalytic activity strictly depending upon the existence of Mn [13]. In vitro experimental evidence further supports the antioxidant function of Mn. After adding 30–180 nM of Mn^2+^ to the cell culture medium, the activity of Mn-SOD in the cells increased, the ROS level decreased, and the redox state of cells improved [14]. Mn can also serve as an effective antioxidant for semen samples in assisted reproductive technology (ART), to prolong the activity and quality of semen [12]. In vivo animal studies further demonstrate that dietary supplementation with Mn enhances the antioxidant capacity of animal tissues. Dietary Mn supplementation significantly upregulates MnSOD expression in poultry embryos, thereby enhancing their antioxidant capacity to counteract the elevated embryonic mortality induced by maternal heat stress [15]. Conversely, mice fed a Mn-deficient diet show a marked elevation in mitochondrial free radical levels, which is significantly and inversely correlated with the activity of the antioxidant enzyme Mn-SOD2 [16].

Numerous studies have demonstrated that oxidative stress is closely linked to the progression of various pathological conditions, including male infertility [17]. The testis, as the primary site of spermatogenesis, is particularly vulnerable to oxidative damage due to its high content of polyunsaturated fatty acids and elevated levels of ROS [18]. ROS exhibit a dual role in testicular physiology. Low concentrations of ROS are essential regulators of key physiological processes such as sperm capacitation, hyperactivation, and the acrosome reaction. However, excessive ROS production can trigger lipid peroxidation and DNA damage, ultimately impairing testicular function [19]. Furthermore, sperm cells are especially sensitive to high ROS levels and are prone to structural and functional damage. Oxidative stress resulting from excessive ROS not only induces DNA fragmentation in sperm but also suppresses enzymatic activity and promotes oxidative protein modifications, which collectively contribute to male infertility [20,21]. Therefore, maintaining the redox balance in the testicular environment through the free radical scavenging (antioxidant) system is crucial for normal testicular function.

The testis, as the core organ of the male reproductive system, produces sperm to maintain fertility. Additionally, it also synthesizes androgens such as testosterone, which regulate sexual development, sustain libido, promote the formation of secondary sexual characteristics, and exert systemic effects on metabolic processes and overall health. Previous research has demonstrated that Mn deficiency in male animals leads to infertility and a lack of sexual desire, accompanied by impaired testicular function, sperm deficiency, degeneration of the seminal ducts, and degeneration of epididymal cells [9]. However, the underlying mechanisms by which Mn deficiency affects testicular development and function remain poorly understood. Given that Mn serves as an essential cofactor for several antioxidant enzymes and participates in the regulation of oxidative stress, this study established a Mn-deficient mouse model and used the interaction between Mn and oxidative stress as a starting point to investigate the mechanisms through which Mn deficiency influences testicular development and function.

## 2. Materials and Methods

### 2.1. Animals and Treatments

Some 45 3-week-old male ICR mice were obtained from Dashuo Biological Technology Company (Chengdu, China) and housed in a carefully sterilized animal facility which was maintained at a relative humidity of 55 ± 5% and a temperature of 25 ± 2 °C. The mice were randomly divided into three groups, namely, the control, Mn-deficient (MnD), and MnD + MnCl_2_ (MnD + Mn) groups. The mice in the control group were maintained on a standard pellet diet containing the normal Mn requirement for 4 weeks and received intraperitoneal injections of normal saline during the final week of the study. Mice in the MnD and MnD + Mn groups received a Mn-deficient diet for 4 weeks and then were separately intraperitoneally injected with saline and MnCl_2_ in the last week. MnCl_2_ (4 mg/kg) injection dosing referenced to Lee et al. [22]. The diets were bought from SPF Biotechnology Co., Ltd. (Beijing, China), with sufficient nutritional requirements according to the AIN-93M Maintenance Purified Diet, a recommended maintenance diet for rodents by the American Institute of Nutrition. All animal experiments were conducted following the National Research Council’s Guide for the Care and Use of Laboratory Animals and were approved by the Animal Care and Use Committee of Southwestern University of Science and Technology (No: LX202300521).

### 2.2. Testis Index

Mice were weighed weekly, and at the end of the feeding experiment, all mice were sacrificed by cervical decapitation under isoflurane, and testes were removed, weighed, and photographed. Testis index was calculated by testis absolute weight (mg) divided by body weight (kg) and multiply 100%.

### 2.3. Histopathological Observation

Testis was collected and fixed with 4% paraformaldehyde. This was followed by dehydration with gradient ethanol and paraffin embedment of the samples. Then, the samples were cut into sections 5 μm thick and H&E-stained. A light microscope (Leica DM500, Heerbrugg, Switzerland) was adopted to observe the histopathological changes.

Histopathological evaluation of testicular tissue damage and spermatogenesis was conducted using Johnsen’s testicular scoring system [23]. The criteria are defined as follows: 10 = complete spermatogenic structures with intact seminiferous tubules; 9 = presence of numerous mature sperm but with disorganized spermatogenic architecture; 8 = only a few sperm present; 7 = absence of sperm but presence of numerous spermatocytes; 6 = only a few spermatocytes present; 5 = absence of sperm and spermatocytes but presence of numerous spermatogonia; 4 = only a few spermatogonia present; 3 = presence of only a few spermatogonial cells; 2 = absence of germ cells while existence of Sertoli cells; 1 = absence of both Sertoli and germ cells.

### 2.4. Mn Content of the Testis

Mouse testis tissues were obtained following the administration of the experimental treatment. Subsequently, 0.5 g of each sample was subjected to wet digestion in heat-resistant test tubes containing a mixture of 1 mL HClO_4_ and 9 mL HNO_3_ (supplied by Chengdu Kelong Chemical Co., Ltd., Chengdu, China) over a 24 h period. The digested samples were then heated on a hot plate until complete evaporation of the liquid occurred. The resulting residue was dissolved in a 10 mL HNO_3_ solution of 0.1 M. Finally, a flame atomic absorption spectrophotometer (AAS700, PerkinElmer, Waltham, MA, USA) was utilized to determine the Mn concentration in prepared samples.

### 2.5. Sperm Quality Assessment

The method for sperm quality detection was based on the research of Yang et al. [24]. After dissection of the mice, the bilateral epididymides were quickly removed and placed in a Petri dish containing 1 mL of BWW culture medium, then rapidly minced. The mixture was incubated at 37 °C for 30 min to form a sperm suspension. After being placed on a sperm counting chamber, 20-μL sperm suspension was analyzed using a sperm analysis system with the assistance of a CASA computer to determine the sperm density and motility in the epididymis. The entire test was completed within 3 min. At least 10 fields of view were examined in each counting chamber.

Eosin staining was performed to evaluate sperm morphology. After spreading 20-μL sperm suspension on a glass slide, it was allowed to be air-dried. Then, the slide was fixed in 4% paraformaldehyde solution for 5 min and air-dried again. A 2% eosin solution was used to stain the sample for 1 h. After staining, the excess dye was washed off and the sample examined under an optical microscope (Leica DM500, Heerbrugg, Switzerland). Sperm malformations involved head defects (double, large, and broken heads), neck curvature, midpiece defects (asymmetry), and principal piece defects (short tails, double tails, irregular bends, and curls), as well as abnormally large cytoplasmic droplets. The sperm malformation rate of each sample was assessed based on the observation of at least 200 sperm.

### 2.6. Evaluation of Oxidative Stress in Testis

Testicular tissue was mixed with an appropriate volume of ice-cold physiological saline and homogenized to prepare a 10% (*w*/*v*) tissue homogenate. The homogenate was then centrifuged at 3000 rpm for 10–15 min, and the resulting supernatant was collected for subsequent analysis. The levels of malondialdehyde (MDA), glutathione (GSH), catalase (CAT), Mn-SOD, and total antioxidant capacity (T-AOC) were measured using commercial assay kits purchased from Nanjing Jiancheng Bioengineering Institute (Nanjing, China).

### 2.7. Quantitative Real-Time PCR (qRT-PCR)

The Trizol reagent was adopted to extract RNA from about 50 mg of mouse testicular tissue, stored in liquid nitrogen. The isolated RNA was then subject to reverse transcription into complementary DNA (cDNA) following the guidelines provided with the reverse transcription kit and preserved at −20 °C for further use. Real-time quantitative PCR was carried out to amplify the target genes, including *Occludin*, *Claudin-11*, *zonula occludens-1* (*ZO-1*), *nuclear factor-erythroid 2-related factor 2* (*Nrf2*), *Kelch-like ECH-associated protein 1* (*Keap1*), *junctional adhesion molecule-A* (*JAM-A*), and *heme oxygenase-1* (*HO-1*), using SYBR Green Master Mix following the manufacturer’s instructions. β-actin was utilized as the internal control, and Table 1 lists the corresponding primer sequences. The 2^−ΔΔCt^ method was used to calculate the relative mRNA expression levels.

### 2.8. Immunofluorescent Staining

The paraffin-embedded mouse testicular tissues were sectioned into 5 μm thick slices, followed by dewaxing and gradient rehydration. Antigen retrieval was then carried out using a citrate-based antigen retrieval solution in a microwave oven. The sections were subsequently cooled to room temperature, rinsed with PBS, and dried. A goat serum-based blocking solution was applied for 30 min, after which it was carefully removed. Subsequently, the diluted primary antibodies ZO-1 (1:2000) and Occludin (1:500) were added, and the sections were incubated overnight at 4 °C. The next day, the sections were brought to room temperature, washed three times, and incubated with fluorescently labeled secondary antibodies. This incubation was performed in the dark at room temperature for 30 min. The sections were then immersed in a fluorescent staining solution for 10 min to enhance signal detection. Nuclei were counterstained with DAPI for 10 min in the dark. After being mounted with coverslips, the sections were examined using a fluorescence microscope (Nikon D-Eclipse C1, Minato, Tokyo, Japan). Finally, the fluorescence intensity was quantitatively analyzed using ImageJ2 software (http://imagej.net/Downloads).

### 2.9. Western Blotting

After thawing the mouse testicular tissues stored in liquid nitrogen, these tissues were homogenized in a RIPA lysis buffer that contained phosphatase and protease inhibitors for total protein extraction. A BCA protein assay kit (P0010S, Beyotime Technology, Shanghai, China) was used to measure the protein concentration. An amount of 30 μg of total protein was combined with loading buffer, denatured by boiling, and separated via sodium dodecyl sulphate-polyacrylamide gel electrophoresis (SDS-PAGE) (4–15%) (P0818S, Beyotime Technology, Shanghai, China). This was followed by the transfer of the resolved proteins onto a PVDF membrane. After blocking the membrane for 1 h at the room temperature, primary antibodies targeting ZO-1 (1:3000 dilution; catalog No. A0659, Abclone, Wuhan, China), Occludin (1:1000 dilution; catalog No. A2601, Abclone, Wuhan, China), JAM-A (1:5500 dilution; catalog No. A2601, Abclone, Wuhan, China), Nrf2 (1:1000 dilution; catalog No. ab137550, Abcam, Cambridge, UK), Keap1 (1:1000 dilution; catalog No. ab119403, Abcam, Cambridge, UK), HO-1 (1:1000 dilution; catalog No. ab68477, Abcam, Cambridge, UK), and β-actin (1:10000 dilution; catalog No. AC026, Abclone, Wuhan, China) were used for incubation overnight at 4 °C. After being washed three times using PBST, the membrane was subject to room-temperature treatment with the HRP-conjugated secondary antibody (1:5000 dilution) for 1.5 h. Following additional washing, an ECL chemiluminescence reagent (P0018A, Beyotime Technology, Shanghai, China) was employed to detect the protein bands. The protein bands were subsequently quantified using ImageJ2 (http://imagej.net/Downloads), with normalization of the relative expression level to β-actin by calculating the gray value ratio of the target proteins to the internal control.

### 2.10. Statistical Analysis

Data, in the form of mean ± standard deviation, were analyzed by variance analyses (LSD or Dunnett’s T3). SPSS software version 22.0 (IBM Corp, Armonk, NY, USA) for windows was utilized to perform all statistical analyses. Differences were regarded as significant at *p* < 0.05.

## 3. Results

### 3.1. Effects of Mn Deficiency on the Body Weight, Testicular Index and Testicular Mn Content in Mice

**Figure 1:**
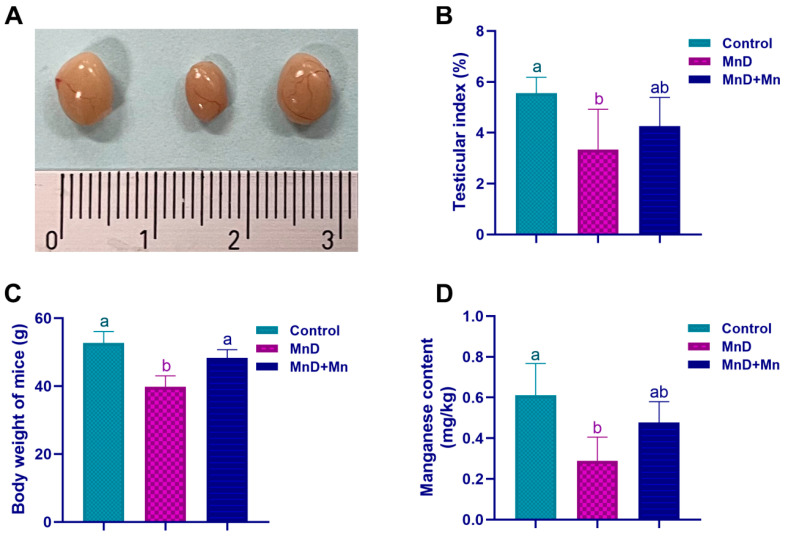
Effect of Mn deficiency on body weight, testicular index and testicular mn content in mice. Notes: (**A**) testis macrostructure; (**B**) testicular index; (**C**) the body weight changes; (**D**) The Mn content. Data are presented with the mean± standard deviation (*n* = 6). ^ab^ Different letters represent significant difference (*p* < 0.05) within the column, and the same letters represent no significant difference (*p* > 0.05).

As shown in Figure 1A, the testis in the MnD group was significantly atrophied and smaller in volume compared to the other groups. Figure 1B,C demonstrate that the testis index and body weight of mice in the MnD group were significantly decreased (*p* < 0.05) compared to the control group, but they recovered to a certain degree after adding MnCl_2_.

Detection results of the testicular Mn content are shown in Figure 1D. The testicular Mn content in the MnD group was significantly lower than that in the control (*p* < 0.05). The testicular Mn content increased to a certain extent relative to the MnD group after supplementation of MnCl_2_.

### 3.2. Effect of Mn Deficiency upon Testicular Histopathology

**Figure 2:**
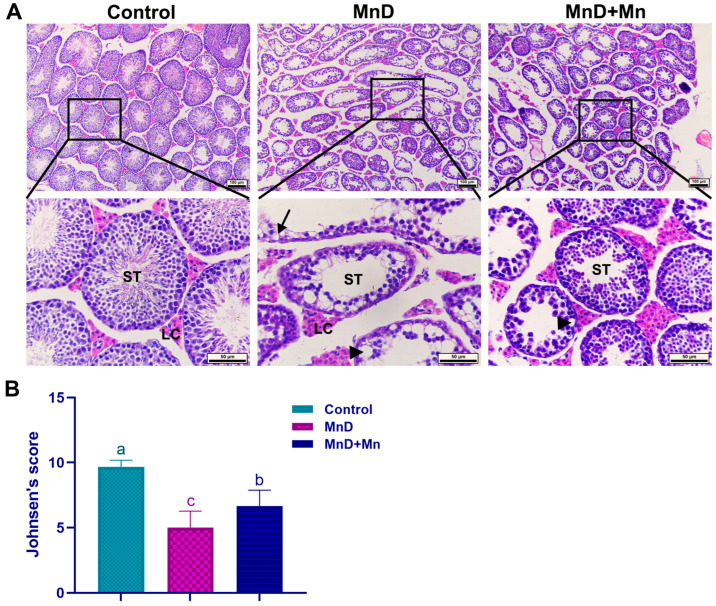
Effect of Mn deficiency on testicular histopathology. Notes: (**A**) the histopathological changes in the testis (HE staining, scale bar = 10 or 50 µm), seminiferous tubule (ST), leydig cell (LC), vacuoles in the cytoplasm (→), vacuoles between cells (►); (**B**) Johnsen’s score. Data are presented with the mean ± standard deviation (*n* = 6). ^abc^ Different letters represent significant difference (*p* < 0.05) within the column.

The histopathological observation results are illustrated in Figure 2A. In comparison with the control, seminiferous tubules in testes of the MnD group presented obvious lesions, including disordered spermatogenic epithelium, reduced numbers of spermatogenic cells at all stages, decreased epithelial thickness, vacuoles between spermatogenic cells and in the cytoplasm, and a reduction or even disappearance of sperm in the lumen. The pathological damage in the testes of the MnD + Mn group was alleviated compared to the MnD group.

The testicular Johnsen’s score is shown in Figure 2B. The MnD group was a significantly lower testicular Johnsen score than the control group (*p* < 0.05). The MnD + Mn group had a significantly higher Johnsen’s score than the MnD group (*p* < 0.05).

### 3.3. Effects of Mn Deficiency upon Sperm Quality in Mice

**Figure 3:**
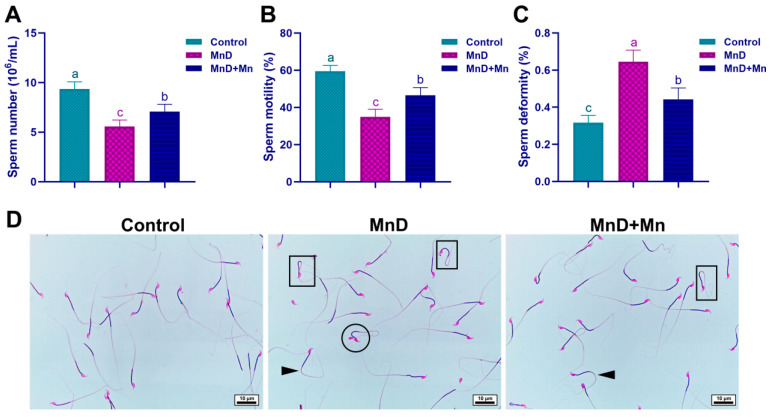
Effects of Mn deficiency on sperm quality in mice. Notes: (**A**) Sperm number; (**B**) Sperm motility; (**C**) Sperm deformity; (**D**) Sperm morphology (Eosin staining, Bar = 10 μm), bent neck (☐), double-headed sperm (⭘), bent principal piece (►). Data are presented with the mean ± standard deviation (*n* = 6). ^abc^ Different letters represent a significant difference (*p* < 0.05) within the column.

As can be seen from Figure 3A–C, the sperm number and sperm motility in the MnD group were significantly lower than those in the control group (*p* < 0.05), while there was a significantly increased sperm malformation rate (*p* < 0.05). After supplementing MnCl_2_, changes in the above indicators were alleviated to a certain extent.

The results of sperm staining were displayed in Figure 3D. The MnD group has a significantly larger number of malformed sperm than the control, with sperm malformations mainly shown as various morphological defects, such as double-headed, neck-bent, midpiece-bent and principal piece-bent. After supplementation with MnCl_2_, the number of abnormal sperm reduced.

### 3.4. Effect of Mn Deficiency on the Blood–Testis Barrier

**Figure 4:**
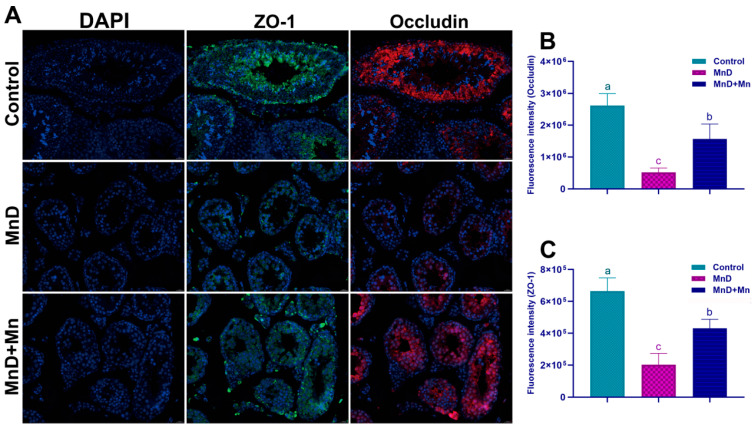
Effect of Mn deficiency on the expression and distribution of blood–testis barrier proteins ZO-1 and Occludin. Notes: (**A**) Results of immunofluorescence staining for ZO-1 and Occludin (Bar = 0.050 mm); (**B**) The fluorescence intensity of ZO-1; (**C**) The fluorescence intensity of Occludin. Data are presented with the mean ± standard deviation (*n* = 6). ^abc^ Different letters represent a significant difference (*p* < 0.05) within the column.

As shown in Figure 4, the immunofluorescence staining results of ZO-1 and Occludin indicated that the fluorescence intensities of ZO-1 and Occludin in the seminiferous tubules of the testis in the MnD group were significantly weakened compared with the control group (*p* < 0.05). Compared with the MnD group, the fluorescence intensities of ZO-1 and Occludin were significantly enhanced after supplementation with MnCl_2_ (*p* < 0.05).

**Figure 5:**
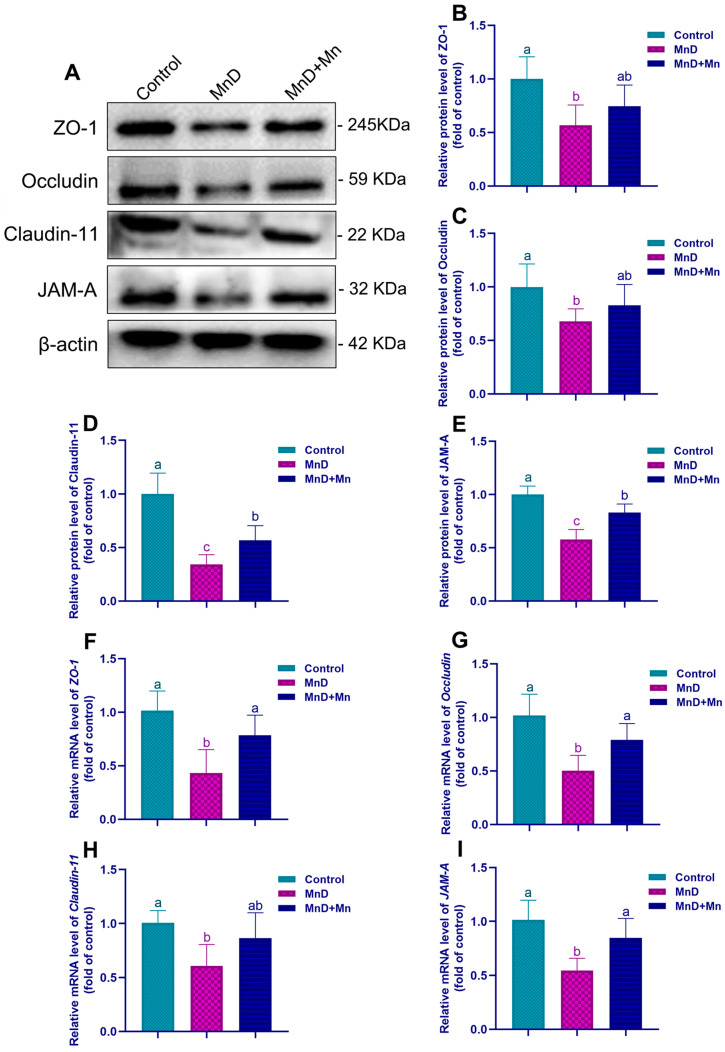
Effect of Mn deficiency on the protein and mRNA expression levels of blood–testis barrier proteins. Notes: (**A**) The representative band pictures of blood–testis barrier proteins; (**B**–**E**) The protein expression of ZO-1, Occludin, Claudin-11 and *JAM-A* (relative to β-actin); (**F**–**I**) The mRNA expression of *ZO-1*, *Occludin*, *Claudin-11* and *JAM-A* (relative to *β-actin*). Data are presented with the mean ± standard deviation (*n* = 6). ^abc^ Different letters represent a significant difference (*p* < 0.05) within the column, and the same letters represent no significant difference (*p* > 0.05).

According to Western blot and qRT-PCR analyses (Figure 5), the MnD group exhibited dramatically down-regulated protein and mRNA expression levels of ZO-1, Occludin, JAM-A, and Claudin-11 compared to the control (*p* < 0.05). In contrast, the protein and mRNA expression levels of these markers in the MnD + Mn group were significantly higher than those in the MnD group (*p* < 0.05).

### 3.5. Effect of Mn Deficiency upon Testicular Oxidative Stress of Mice

**Figure 6:**
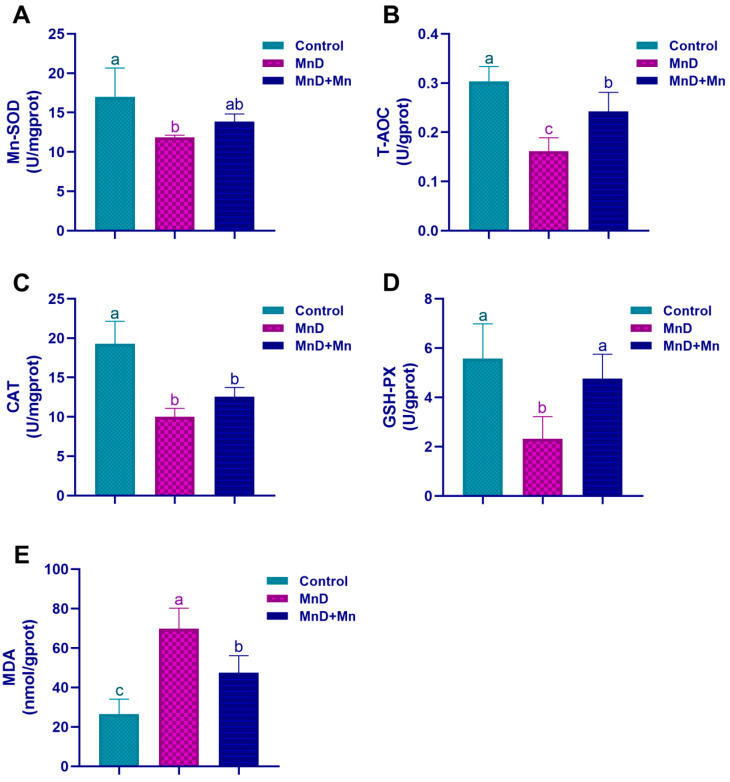
Effects of Mn deficiency on oxidative stress in the mice. Notes: (**A**) Mn-SOD, (**B**) T-AOC, (**C**) CAT, (**D**) GSH-PX, (**E**) MDA. Data are presented with the mean ± standard deviation (*n* = 6). ^abc^ Different letters represent a significant difference (*p* < 0.05) within the column, and the same letters represent no significant difference (*p* > 0.05).

To evaluate the impact of Mn deficiency on testicular oxidative stress of mice, the levels or activities of Mn-SOD, T-AOC, GSH-Px, CAT, and MDA were analyzed. As illustrated in Figure 6, Mn deficiency led to a significant reduction in T-AOC levels and CAT, Mn-SOD, and GSH-Px activities, while markedly increasing MDA levels in the testis (*p* < 0.05). Nevertheless, these adverse effects were notably mitigated after supplementation with MnCl_2_.

### 3.6. Effect of Mn Deficiency on the Nrf2 Pathway in the Testis of Mice

**Figure 7:**
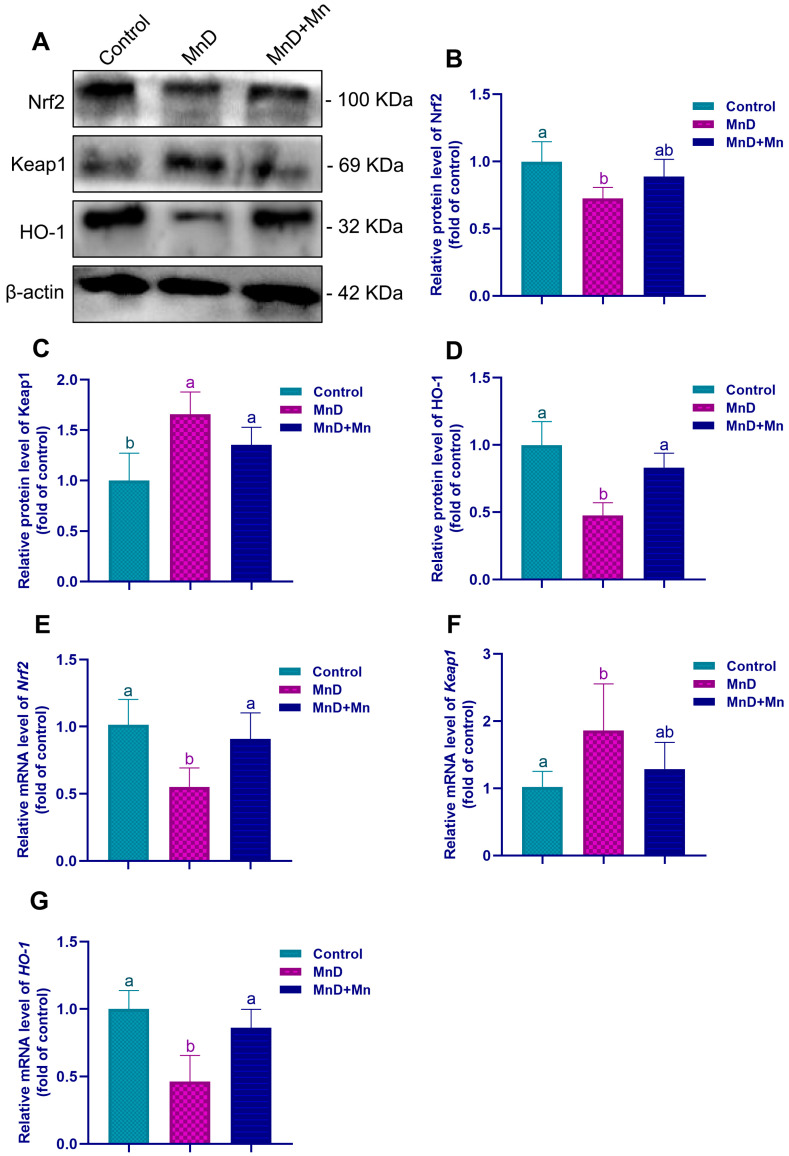
Effects of Mn deficiency on the Nrf2 pathway in the testes of mice. Notes: (**A**) The representative band pictures of Nrf2 pathway-related molecules; (**B**–**D**) The protein expression of Nrf2, Keap1 and HO-1 (relative to β-actin); (**E**–**G**) The mRNA expression of *Nrf2*, *Keap1* and *HO-1* (relative to *β-actin*). Data are presented with the mean ± standard deviation (*n* = 6). ^abc^ Different letters represent a significant difference (*p* < 0.05) within the column, and the same letters represent no significant difference (*p* > 0.05).

Figure 7 illustrates the effects of Mn deficiency upon the expression of key molecules in the Nrf2 pathway in the testis. The MnD group exhibited significantly lower mRNA and protein expression levels of both HO-1 and Nrf2 (*p* < 0.05), whereas the mRNA and protein expression levels of Keap1 were significantly higher (*p* < 0.05) than those of the control. Notably, in the MnD + Mn group, supplementation with Mn significantly restored both the protein and mRNA expression of HO-1 and Nrf2 (*p* < 0.05) relative to the MnD group.

## 4. Discussion

Testicles are crucial reproductive organs in male animals responsible for sperm production and the secretion of male hormones. In this study, mice receiving a Mn-deficient diet exhibited a significant reduction in the Mn concentration in testicular tissues after five weeks. Furthermore, Mn deficiency resulted in reduced testicular volume, a lower testicular index, and structural damage to testicular tissue. Histopathological examination revealed that Mn deficiency in mice resulted in disorganization of the spermatogenic epithelium within seminiferous tubules, decreased number of spermatogenic cells at all developmental stages, decreased epithelial thickness, the presence of vacuoles between spermatogenic cells and within their cytoplasm, and a significantly lower Johnsen’s score in the testis. However, after one week of Mn supplementation, the reduction in testicular index and the histopathological damage of testicular tissue caused by Mn deficiency were alleviated. The aforementioned results demonstrate that Mn is vital for the normal development of testis. Mn deficiency can impair testicular development and induce structural abnormalities in the seminiferous tubules, whereas Mn supplementation can mitigate these adverse effects. Currently, reports on the impact of Mn deficiency on testicular development and histomorphological changes remain limited. Earlier studies have yielded findings consistent with those of the present study. For instance, Boyer et al. [25] reported that Mn deficiency in male rats results in testicular atrophy and complete infertility due to impaired spermatogenesis.

Sperm density, motility and abnormality rate detection are rapid and effective methods for evaluating the reproductive development of male animals [24]. This study reveals that Mn deficiency in mice significantly reduces sperm density and motility while markedly increasing the sperm deformity rate. These alterations in sperm quality parameters correspond to the previously observed pathological changes in the testicular germinal epithelium and the disruption of spermatogenesis associated with Mn deficiency. Alterations in sperm morphology can serve as a direct indicator of their developmental status. Our findings reveal that Mn deficiency significantly elevates the incidence of sperm malformations, with typical abnormalities including double heads, curved necks, and coiled tails. Spermatogenesis is a complex and highly regulated process through which spermatogonia undergo progressive differentiation and maturation. This study demonstrates that Mn deficiency markedly disrupts spermatogenesis, leading to declined sperm quality and an increased prevalence of morphologically abnormal sperm. Accumulating evidence indicates that semen contains a variety of trace elements, including calcium, Mn, copper, zinc, magnesium, and selenium. These elements are essential for normal sperm production, reproductive health, sperm maturation, motility, capacitation, and normal sperm function. Among them, Mn is an effective stimulant for sperm motility, which can improve the quality of semen and thereby increase the success rates of artificial insemination and in vitro fertilization [26]. Adejuwon et al. [27] found that the serum Mn level in patients with normal sperm counts but unexplained infertility is higher than those with azoospermia or oligospermia. Similarly, Reis et al. [28] reported that supplementing the diet of bulls with an appropriate amount of Mn ions (Mn^2+^) significantly increased the production of morphologically intact sperm, while markedly reducing the incidence of sperm with damaged acrosomes or other structural impairments. Collectively, these findings and the present study demonstrate that Mn serves as a crucial trace element in maintaining sperm quantity, motility, and morphological integrity.

The blood–testis barrier (BTB), as a critical anatomical structure between seminiferous tubules and capillaries within the testis, serves as a key component in maintaining spermatogenesis [29]. The structural integrity of the BTB ensures a stable microenvironment within the testis that is essential for normal spermatogenesis [30,31]. Tight junctions (TJs) represent the largest, most complex, and most functionally significant intercellular structures within the BTB, playing a critical role in selectively regulating substance permeation and keeping homeostasis of the spermatogenic microenvironment of the testis. Main components of TJs include Occludin, Zonula occludens (ZO), Junctional Adhesion Molecule (JAM-A/F11R), Claudins, etc. [32]. Further, to reveal the effect of Mn deficiency upon the BTB, the expression levels of TJ-related proteins were examined. Research results showed that Mn deficiency significantly reduced the fluorescence intensity of ZO-1 and Occludin and affected their distribution. qPCR and WB assays demonstrated that Mn deficiency decreased the mRNA and protein expression levels of ZO-1, Claudin-11, Occludin, and JAM-A. Notably, these adverse effects were alleviated by Mn supplementation, indicating that Mn may play a key role in maintaining the function of the BTB in mice. Moreover, previous studies have demonstrated that excessive Mn exposure can impair the BTB, as indicated by the down-regulation of key BTB biomarkers such as *Occludin*, *Claudin-1*, *ZO-1* and *JAM1* [33]. In contrast, the impact of manganese deficiency on the structure and function of the BTB has not been systematically studied, and there is currently no comprehensive evidence to support it. To further clarify the regulatory role of manganese on the BTB, future research could combine transmission electron microscopy observation with the biotin tracing method for functional verification to make up for the limitations of this study in methodology.

Mn serves as an essential cofactor for numerous biologically active enzymes. Among them, MnSOD is a Mn-dependent metalloenzyme localized in the mitochondrial matrix, where it catalyzes superoxide anion radicals to be dismutated into hydrogen peroxide and molecular oxygen. Consequently, Mn is vital for maintaining the antioxidant defense system of the body. Previous studies have demonstrated that Mn deficiency significantly reduces the activity of critical antioxidases, such as SOD, glutathione reductase (GR), glutathione peroxidase (GPx), and CAT, in the heart, liver, kidneys, and pancreas of rats, thereby inducing the oxidative stress state in the organism [34]. To further investigate the potential involvement of oxidative stress in testicular dysfunction induced by Mn deficiency, this study assessed oxidative stress levels in the testis. It was found that Mn deficiency significantly reduced the activities of Mn-SOD, CAT and GSH-PX in the testis, decreased the level of T-AOC, while the level of MDA significantly increased. However, after Mn supplementation, the changes in the above oxidative stress indicators could be alleviated. Furthermore, accumulating evidence indicates that Mn supplementation enhances testicular antioxidant capacity, mitigates oxidative stress damage induced by formaldehyde (FA), and improves both sperm parameters and testicular morphology in FA-treated mice [35,36]. These findings agree with the above research results, collectively demonstrating that Mn is crucial for enhancing the antioxidant capacity of testes. The Nrf2 signaling pathway serves as a critical regulatory mechanism in cellular responses to oxidative stress. Nrf2 activity is tightly controlled by its inhibitor Keap1, with which it forms a complex in the cytoplasm, thereby maintaining the inactive state of Nrf2. After cells are exposed to certain external stimuli, Keap1 undergoes structural modifications that lead to the release of Nrf2. This enables Nrf2 to translocate into the nucleus, in which Nrf2 binds to antioxidant response elements (ARE), subsequently inducing the expression of antioxidases including SOD, GPx, and CAT [37,38]. This study found that Mn deficiency significantly down-regulated the Nrf2 and HO-1 expression in the testis, and significantly up-regulated the Keap1 expression. However, supplementation with Mn alleviated these variations, suggesting that Mn deficiency can lower the antioxidant capacity by suppressing the Nrf2 signaling pathway, thereby causing testicular oxidative stress. Similarly, Qin et al. [39] demonstrated that Mn mitigates cardiomyocyte apoptosis induced by heat stress by inhibiting ROS generation and activation of the Nrf2/SOD2 signaling pathway, thereby protecting cardiomyocytes during chick embryonic development from oxidative stress. Jiang et al. [40] also reported that Mn deficiency leads to intestinal inflammation and disruption of the intestinal barrier of grass carp, which shows a close association with dysregulation of TOR, NF-κB, and Nrf2 signaling pathways. Integrating these findings, it can be inferred that Mn deficiency may promote oxidative damage in the testis by inhibiting the Nrf2 signaling pathway, thereby impairing testicular development, compromising BTB integrity, and disrupting spermatogenesis.

## 5. Conclusions

In conclusion, our study demonstrates that Mn deficiency leads to structural damage in testicular tissue, reduced sperm quality, BTB dysfunction, and oxidative stress injury. The underlying mechanism may involve the suppression of antioxidant enzyme activity and the inhibition of the Nrf2 signaling pathway, which collectively elevate oxidative stress levels in the testis. This ultimately results in impaired testicular function, disrupted spermatogenesis, and compromised BTB integrity. These findings provide novel experimental evidence that enhances our understanding of the molecular mechanisms underlying reproductive dysfunction induced by Mn deficiency.

## Figures and Tables

**Table 1:** The sequence of primers used in the study.

Gene	Accession Number	Forward (5′→3′)	Reverse (3′→5′)
*ZO-1*	NM_009386.2	GAGCCCCCTAGTGATGTGTG	TAGGGTCACAGTGTGGCAAG
*Occludin*	NM_008756.2	TAGTGGCTTTGGCTACGGAGGT	AGGAAGCCTTTGGCTGCTCTTG
*Claudin-11*	NM_016674.4	CCCTTCAGCAGAGCAAGGTT	TAGGGCAACCAAGTGCCTTT
*JAM-A*	NM_001382727	CAGAGGTGATTCATGGCTCTGTG	TTCCAGGCTGGCAATAACTGAC
*Nrf2*	NM 010902	GCAACTCCAGAAGGAACAGG	AGGCATCTTGTTTGGCAATG
*Keap1*	NM 010442	ACGACGTGGAGACAGAGACC	ATCAATTTGCTTCCGACAGG
*HO-1*	NM_016679	CCAGAGTCCCTCACAGAT	CCCAAGAGAAGAGAGCCA
*β-actin*	NM_007393	GGCTGTATTCCCCTCCATCG	CCAGTTGGTAACAATGCCATGT

## Data Availability

The data presented in this study are available on request from the corresponding author due to privacy restrictions.
